# Analysis of Velocity Profiles and Power Prediction for Single‐Stage Gravitational Vortex Water Turbine (GVWT)

**DOI:** 10.1155/tswj/2200648

**Published:** 2026-09-30

**Authors:** Erna Septyaningrum, Sutardi, Ridho Hantoro, Aurellia Rossa Puspa Maharani

**Affiliations:** ^1^ Department of Engineering Physics Institut Teknologi Sepuluh Nopember Surabaya East Java Indonesia its.ac.id; ^2^ Department of Mechanical Engineering Institut Teknologi Sepuluh Nopember Surabaya East Java Indonesia its.ac.id

**Keywords:** CFD, gravitational vortex water turbine (GVWT), low-head hydropower, power prediction model, velocity profile

## Abstract

The gravitational vortex water turbine (GVWT) is a promising technology for low‐head hydropower applications; however, its internal flow structure and hydrodynamic basis for multistage runner placement remain insufficiently understood. A GVWT primarily consists of an inlet channel, vortex‐forming basin, and runner installed within the vortex‐forming basin. This study focuses on the inlet‐channel notch angle configuration and its influence on the conical basin flow field, single‐stage power prediction, and residual flow criteria for second‐stage runner placement. This study investigated the velocity profiles and power prediction of a single‐stage GVWT in two conical basin configurations, namely, Basin A (conical basin with 0° inlet‐channel notch angle) and Basin B (conical basin with 19° inlet‐channel notch angle), with the aim of deriving design‐relevant guidance for multistage implementation. Computational fluid dynamics (CFD) simulations were performed to examine vortex formation and the distributions of tangential, axial, and radial velocities before and after runner installation. The results show that Basin B produces a more symmetric and vertically coherent vortex, along with a more uniform tangential velocity distribution, smoother axial flow, and reduced radial diffusion compared with Basin A. These characteristics create a favorable hydrodynamic environment for energy extraction. A semiempirical power prediction model was developed using Buckingham pi analysis and evaluated as a practical design tool using experimental data for a 10 cm propeller runner installed at different depths. The model reproduced the experimental trend well, with deviations of approximately 2% across the tested runner depths. Downstream flow analysis further showed that significant tangential kinetic energy remained below the first‐stage runner, indicating that the lower basin retained sufficient hydrodynamic quality for evaluating second‐stage runner placement. Based on the combined velocity and kinetic‐energy distributions, the region *z*/*H* = −0.9 to −0.8 was identified as the most suitable location for second‐stage runner installation because it offers high residual tangential energy, moderate axial energy, reduced radial diffusion, and improved submergence, providing a depth‐based design criterion for multistage GVWT systems. Therefore, this study provides a hydrodynamics‐based design framework for predicting the single‐stage GVWT performance and identifying suitable second‐stage runner placements in multistage systems.

## 1. Introduction

Low‐head hydropower (LHP) has attracted increasing attention for decentralized electricity generation, particularly in rural and remote areas where conventional hydropower systems are often less practical [1, 2]. According to the International Renewable Energy Agency (IRENA), renewable sources are projected to provide up to two‐thirds of the global energy supply by 2050, reflecting their central role in a sustainable energy future [3]. By the end of 2019, renewables accounted for 27% of global electricity generation, with hydropower contributing nearly 20% of total electricity output and more than 80% of renewable electricity production [4–6]. These figures highlight hydropower′s enduring importance not only as a mature renewable technology but also as a cornerstone for sustainable and equitable energy access, particularly in regions with abundant water resources.

LHP has attracted significant attention due to its applicability in small‐scale, decentralized energy systems, particularly in rural and remote areas. Conventional turbines (Francis, Kaplan, and cross‐flow) can operate at low heads (ranging from 3 to 40 m) but often require complex mechanical control and high fabrication precision [7, 8]. Francis turbines are relatively expensive to manufacture due to their compact size and multiple blades [9]. Kaplan turbines are suitable for ultralow head (ULH) applications (0–3 m), but their reliance on blade angle control systems increases cost and maintenance requirements [7, 10]. According to Jang et al. [11] and Morabito et al. [12], LHP turbines also include pump‐as‐turbine (PAT) and gravitational vortex water turbine (GVWT). The GVWT offers a low‐cost, easy‐to‐install solution for low‐head applications, typically operating with heads of 1.5–5 m and flow rates above 1 m^3^/s [13]. Its design simplicity eliminates the need for dams or high civil infrastructure, making it suitable for rural electrification [14]. Among low‐head technologies, the GVWT offers a simple and potentially low‐cost solution because it can operate under relatively low hydraulic heads and requires limited civil infrastructure.

Recent studies have explored the effects of basin geometry, inlet‐channel notch angle, and runner design on GVWT performance. Inlet‐channel notch angle help guide the flow and initiate vortex formation [15, 14, 16] Power et al. [17] reported that curved‐blade runners offer higher efficiency than straight blades. Saleem et al. [18] demonstrated that cylindrical basins combined with optimized runners can achieve efficiencies exceeding 20%. Dhakal et al. [19] conducted an experimental GVWT study using a paddle‐type runner in a cylindrical basin and observed the influence of blade size, number of blades, inlet flow rate, and inlet height, achieving a maximum efficiency of 15.1%. Studies confirm that blade number significantly affects turbine performance [17, 19, 20].

Several innovations have been proposed to improve GVWT performance. Wichian and Suntivarakorn [21] improved the runner design by incorporating baffle plates, resulting in an efficiency of 32.79%. Propeller‐type runners are considered more suitable for low‐head applications due to their simple construction, ease of installation, and low maintenance requirements [22]. Singh and Nestmann [23] evaluated the impact of blade angle on propeller runner performance and achieved 70% efficiency. Wardhana et al. [24] used a Gottingen 428 hydrofoil on propeller runner, reaching 54.4% efficiency. Similarly, Slachmuyders et al. [16] developed a propeller runner design for GVWT with overall system efficiency exceeding 50%. To increase the energy output, multistage GVWT configurations have been proposed, where more than one runner is installed within a single basin [25, 26].

The free vortex formed in the basin has a complex flow structure. Energy from the inflow creates circulation (*Γ*∞), inducing a pressure drop near the outlet and forming an air core. The flow is generally axisymmetric, where tangential velocity decreases with increasing radial distance and varies with depth [27]. Basin flow consists of primary and secondary components, with the latter driven by centrifugal forces that can introduce instabilities [28]. Because the propeller runner relies heavily on flow velocity and direction, detailed understanding of the basin′s internal velocity profiles is essential—especially in multistage systems where residual flow influences downstream runner performance.

Despite this progress, fewer studies have explicitly linked the internal flow structure of GVWT basins to both power prediction and multistage runner placement. In particular, detailed information on the tangential, axial and radial velocity distributions before and after runner installation remains limited. This issue is important because the propeller runner directly depends on the magnitude and direction of the incoming flow. In addition, for multistage configurations, the residual flow downstream of the first runner determines whether sufficient rotational and axial energy remains available for a second‐stage runner. Therefore, a clearer understanding of the basin velocity field is required not only to improve single‐stage performance but also to support design decisions for multistage GVWT systems. This lack of detailed internal‐flow analysis remains one of the main unresolved issues in current GVWT research.

Another practical limitation in GVWT development is the difficulty of estimating turbine power output without conducting full turbine experiments or detailed runner simulations. Although experimental testing provides reliable performance data, it is time‐consuming and costly when many geometric and operating parameters must be evaluated. For this reason, a predictive framework based on measurable or simulated flow quantities would be valuable as a design tool, especially during the preliminary design stage.

The contribution of this work lies in integrating flow‐field analysis and semiempirical modeling to provide a design‐oriented interpretation of basin hydrodynamics for GVWT applications. Specifically, this study (1) compares the hydrodynamic effects of two conical basin configurations, (2) develops and evaluates a semiempirical model for predicting the power output of a single‐stage GVWT, and (3) establishes a hydrodynamic basis for second‐stage runner placement in a multistage configuration using velocity and kinetic‐energy distributions. By linking internal flow characteristics to power prediction and runner placement, this study provides a practical framework for GVWT design under low‐head operating conditions. This design‐oriented approach strengthens the engineering relevance of the present study by connecting basin hydrodynamics with performance prediction and multistage design decisions.

## 2. Methods

### 2.1. GVWT Geometry and Configuration

The GVWT system considered in this study consisted of three main components: an inlet channel, a conical basin, and a propeller runner. Water entered the basin tangentially through a rectangular inlet channel, generating a free‐surface vortex before exiting through the bottom outlet. This configuration was selected because the resulting swirling flow provides the tangential and axial velocity components required to drive the runner and sustain energy extraction within the basin. The geometric arrangement of the single‐stage GVWT used in this work is illustrated in Figure 1.

**Figure 1:**
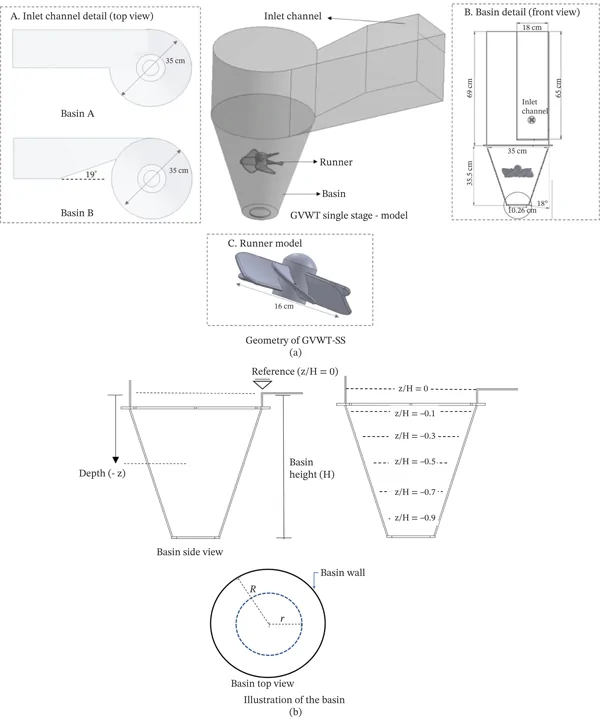
GVWT single stage design. (a) Geometry of GVWT‐SS. (b) Illustration of the basin.

This study investigates two GVWT configurations with identical conical basin geometry but different inlet‐channel notch angles, namely Basin A with a 0° inlet‐channel notch angle and Basin B with a 19° inlet‐channel notch angle, to evaluate their effects on vortex symmetry, vortex height, and internal flow characteristics (Figure 1a). The GVWT system includes a rectangular inlet channel, a conical basin, and a propeller runner positioned at mid‐depth, as recommended by Singh and Nestmann [23]. The runner was placed at 50% of the basin depth based on prior findings indicating that this region contains relatively high tangential velocity suitable for energy extraction. The inlet‐channel notch angle in Basin B is selected based on previous experimental studies [29] In that study, the 19° notch‐angle configuration was shown to promote a more axisymmetric vortex structure and improve flow alignment near the inlet region. Therefore, the present work uses this previously validated inlet‐channel configuration to further examine its influence on internal velocity distribution, power prediction, and hydrodynamic suitability for multistage GVWT development.

The basin had a diameter of 35 cm and a total height of 105 cm, whereas the bottom outlet diameter was 10.26 cm. These dimensions were used consistently for all simulated cases. The conical basin geometry was selected because it is commonly used in GVWT systems to promote vortex formation and guide the swirling flow toward the outlet.

Two basin configurations were investigated to evaluate the influence of inlet modification on vortex formation and internal flow behavior. The first configuration, referred to as Basin A, was a conical basin inlet‐channel notch angle of 0°. The second configuration, Basin B, included a 19° inlet‐channel notch angle installed near the inlet region. This geometry was adopted to improve the alignment of the incoming flow and promote a more axisymmetric vortex structure. The comparison between these two basins was intended to clarify how inlet conditioning affects vortex symmetry, vortex height, and the distribution of tangential, axial, and radial velocities inside the basin.

To evaluate the effect of geometry on basin hydrodynamics, two simulation scenarios were considered for each configuration. In the first scenario, the basin was analyzed without a runner to characterize the natural vortex formation. In the second scenario, the single‐stage runner was installed to assess its influence on the internal flow structure and on the residual flow below the runner plane. This approach made it possible to distinguish the effect of inlet geometry from the effect of runner installation and to identify the flow region that remains potentially useful for a second‐stage runner in a multistage GVWT system. The five‐blade propeller runner was selected based on the authors′ previous experimental study on GVWT runner design, which showed that four‐blade and five‐blade runners provided better performance than three‐blade and six‐blade runners. Although the favorable single‐stage installation depth was found to be around 60%–70% of the basin depth, the runner in the present computational fluid dynamics (CFD) analysis was positioned at (*z*/*H* = −0.5) as a baseline first‐stage location to provide sufficient downstream space for a second‐stage runner in a multistage GVWT configuration. Therefore, the (*z*/*H* = −0.5) position should not be interpreted as the universal optimum for single‐stage operation, but as a design choice for evaluating residual flow and second‐stage runner placement [30].

The runner consisted of five blades and was positioned at 50% of the basin depth. This location was chosen because previous observations indicated that the mid‐depth region contains a relatively strong tangential velocity component, making it suitable for energy extraction. The propeller‐type runner was selected because it offers simple construction and has shown favorable hydraulic performance for low‐head GVWT applications.

For the flow analysis, the basin was divided into several observation planes along the vertical direction. The reference position *z*/*H* = 0 was defined at the base level of the inlet channel, and the same coordinate convention was used for both the empty‐basin and runner‐installed cases. Velocity data were extracted at several depths to compare the flow structure on the left and right sides of the basin and to evaluate the degree of vortex symmetry produced by each configuration. This geometric framework was then used consistently throughout the CFD analysis, the power prediction model development, and the assessment of the candidate location for the second‐stage runner.

### 2.2. Numerical Model

CFD simulations were performed using ANSYS CFX to analyze the internal flow structure of the GVWT basin and to quantify the velocity field associated with vortex formation and runner installation. CFD simulation has been used in several studies to predict flow profiles [19, 31–33]. The numerical model was developed to resolve the free‐surface vortex inside the conical basin and to extract the tangential, axial, and radial velocity components at several vertical locations. Two simulation scenarios were considered for each basin configuration. In the first scenario, the basin was analyzed without a runner to characterize the natural vortex structure generated by the incoming tangential flow. In the second scenario, a single‐stage propeller runner was installed to evaluate its influence on the velocity distribution and on the residual flow available below the runner plane. This approach made it possible to distinguish the effect of basin geometry from the effect of runner operation and to provide the hydrodynamic basis for the subsequent power prediction and multistage design analysis.

The simulations were conducted under steady‐state conditions. This assumption was adopted to obtain the mean flow characteristics inside the basin, which were required for comparing the two basin configurations and for developing the semiempirical power prediction model. The convergence criterion was defined by a residual target of 10^−4^, and the solution was considered converged when all governing equations satisfied this criterion and the monitored flow variables no longer exhibited significant variation.

The air–water flow inside the basin was modeled using an Eulerian multiphase approach with the volume of fluid (VOF) method. This method was selected because it is suitable for capturing the free‐surface interface and the air core formed in a gravitational vortex. In the present work, the free surface was represented by the water volume fraction distribution, and the interface was identified at a volume fraction of 0.5 [34, 18, 35].

A preliminary turbulence‐model comparison was conducted between the RNG *k* − *ε* and *k* − *ω* SST models. The RNG *k* − *ε* model was selected because it reproduced the free‐surface vortex shape and falling‐water curvature more consistently with the experimental observation while also providing stable convergence at reasonable computational cost. Although the Reynolds stress model may provide a more detailed representation of turbulence anisotropy, it was not used in this design‐oriented study because of its higher computational cost and convergence demand. In the present case, the RNG *k*‐*ε* model was considered appropriate for representing the strong rotational motion and the flow gradients that develop near the vortex core and the outlet region [34, 36, 37]. The inlet boundary was specified as a mass‐flow inlet with a flow rate of 0.0121 m^3^/s, while the upper boundary of the basin and the outlet were treated as opening boundaries. This configuration allowed water to enter the domain through the inlet channel at a prescribed flow rate, whereas the upper free surface and the downstream outlet remained open to the surrounding atmosphere. The same boundary condition scheme was applied to all cases in order to ensure a consistent basis for comparing Basin A and Basin B, both with and without the runner. The boundary conditions set up is given in Figure 2.

**Figure 2:**
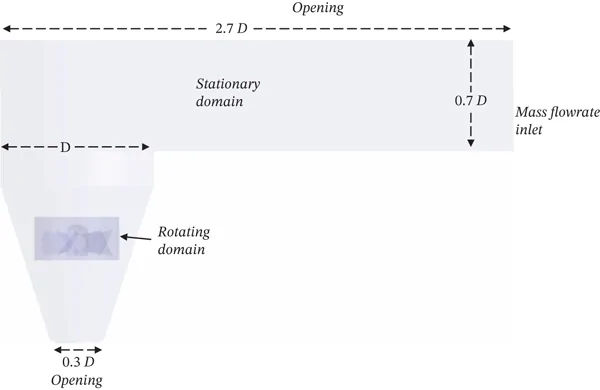
Boundary conditions for CFD simulation.

To represent the rotor motion, the runner was modeled using a rotating domain approach. This method was adopted to account for the interaction between the rotating runner and the surrounding swirling flow without introducing unnecessary computational complexity. By applying the rotating domain treatment, the effect of runner installation on the local velocity field and on the downstream residual energy could be evaluated more realistically than with a stationary runner assumption.

The computational mesh was generated using a hybrid discretization strategy. The conical basin was discretized mainly using tetrahedral elements, whereas additional refinement was applied in regions with large velocity gradients, particularly near the runner. To improve the mesh resolution around the runner blades, an inflation‐layer treatment was applied with a first‐layer thickness of 0.67 mm and a growth rate of 1.2. The mesh contained approximately 2.25 × 10^5^ nodes for the empty‐basin simulation and approximately 4.3 × 10^5^ nodes for the basin‐with‐runner simulation. The maximum skewness of the generated mesh was 0.88, indicating that the mesh quality remained within the acceptable range for the present CFD calculation. The evaluated *y* ^+^ value on the runner blade surface and basin wall were 17.25 and 60.2, respectively.

Consisting primarily of tetrahedral elements in the basin region and quadrilateral elements in the inlet channel, this combination was selected to accommodate the geometric complexity of the conical basin while reducing the mesh density in less critical regions, thereby improving computational efficiency. Greater attention was given to the basin and outlet regions, where the strongest gradients in velocity and free‐surface deformation were expected.

For the flow analysis, the basin was divided into several observation planes along the vertical direction, as illustrated in Figure 1b. The reference position *z*/*H* = 0 was defined at the base level of the inlet channel, and the same coordinate system was used consistently in all cases. Velocity components were extracted from both the left and right sides of the basin at identical nondimensional depths in order to evaluate vortex symmetry and to compare the flow structures of the two basin configurations. This procedure was applied to both the empty‐basin and runner‐installed cases.

The principal outputs obtained from the CFD model were the free‐surface vortex profile and the distributions of tangential, axial, and radial velocities. These results were used for three main purposes. First, they were used to assess the effect of the 19° inlet‐channel notch angle on vortex symmetry and basin hydrodynamics. Second, they were used to quantify the changes in flow structure caused by the installation of the single‐stage runner. Third, the extracted velocity data were used as the basis for the semiempirical power prediction model and for identifying the depth range that remains potentially suitable for the placement of a second‐stage runner in a multistage GVWT system.

### 2.3. Grid Independent Test and Model Validation

A grid independence test was carried out to ensure that the numerical results were not significantly affected by mesh density, as shown in Figure 3. Five mesh levels were evaluated, and the final grid was selected when the variation in the monitored variable became smaller than 3%. The monitored parameter for the grid independence test was average outlet velocity, and the final mesh was selected because further refinement produced only negligible change in the numerical results.

**Figure 3:**
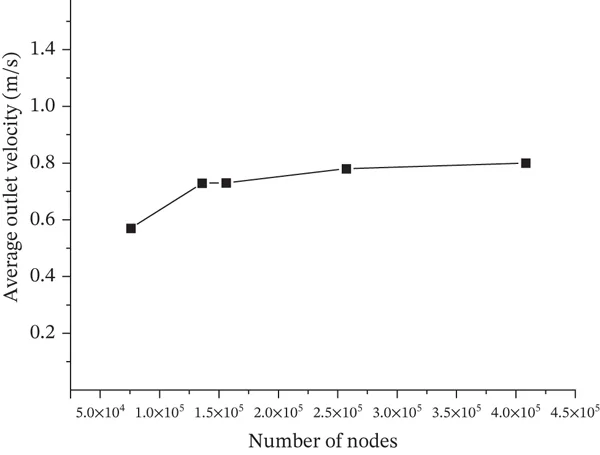
Grid independence test.

The numerical model was validated by comparing the predicted vortex height with experimental measurements under three discharge conditions. As presented in Table 1, the simulated vortex heights were 35.5, 41.0, and 46.5 cm at flow rates of 0.0056, 0.0081, and 0.0121 m^3^/s, respectively, whereas the corresponding experimental values were 35.8, 43.5, and 51.0 cm. The resulting deviations were 1%, 6%, and 9%, with an average deviation of 5%. This level of agreement indicates that the CFD model is capable of reproducing the free‐surface vortex behavior with satisfactory accuracy. Therefore, the validated numerical setup was considered appropriate for the subsequent analysis of vortex characteristics, velocity distributions, and hydrodynamic conditions relevant to power prediction and multistage runner placement.

**Table 1:** Comparison of experimental and simulated vortex heights for model validation.

No	Flowrate (m^3^/s)	Vortex height (cm)		Deviation
		Experiment	Simulation	
1	0.0056	35.8	35.5	1%
2	0.0081	43.5	41	6%
3	0.0121	51	46.5	9%
Average deviation				5%

Beyond the macroscopic validation of the free‐surface profile, the accuracy of the internal velocity field is indirectly substantiated through the performance of the semiempirical power prediction model. The model′s formulation relies fundamentally on the tangential and axial velocity components extracted from the CFD‐derived flow field. Given that the predicted power shows close agreement with experimental measurements—achieving a low deviation of approximately 2%—it can be inferred that the simulated internal hydrodynamic distributions are physically representative of the actual vortex behavior. This indirect validation provides additional confidence in the reliability of the numerical results for evaluating the basin′s energy potential and informing multistage runner placement.

### 2.4. Semiempirical Power Prediction Formulation

A semiempirical formulation was developed to estimate the power output of the single‐stage GVWT based on the hydrodynamic characteristics of the basin flow. The purpose of this formulation was to provide a practical predictive tool that links the internal vortex behavior to turbine performance without requiring a full simulation for every design condition. In this study, the formulation was established using Buckingham pi analysis to identify the nondimensional relationship between turbine power and the governing flow and geometric parameters.

In the general dimensional formulation, the power output of a GVWT may depend on the fluid properties, flowrate, runner geometry, and basin geometry. The model relates the power output to key physical variables, including flow rate (*Q*), runner depth (*z*), diameter where the runner placed (*D*_*b*_(*z*)), runner diameter (*D*_*r*_), dynamic viscosity (*μ*), fluid density (*ρ*), and angular velocity (*ω*). Based on dimensional analysis, the governing relationship was expressed in the following nondimensional form:

(1) PDr4ρQ3=fμρωDr2,ZDr,DbzDr

The formulation was then used to construct a predictive relationship between the nondimensional parameters and the turbine power. The required variables were obtained from the CFD‐derived flow field, whereas the predictive capability of the resulting model was evaluated using experimental data. In this way, the proposed formulation served as an intermediate design tool between CFD‐based hydrodynamic analysis and experimental performance assessment.

To evaluate its predictive capability, the power estimated by the semiempirical model was compared with the corresponding experimental power data for a 10 cm propeller runner installed at different depths. The comparison showed deviations of about 2%, as given in Table 2.

**Table 2:** Comparison of experimental and model prediction for GVWT power.

Runner position	Power (watt)		
	Experiment	Prediction	Error
−0.6	2.49	2.418	3%
−0.7	3.20	3.21	0%
−0.8	3.71	3.552	4%
Average error			2%

## 3. Results and Discussion

### 3.1. Basin‐Induced Vortex Characteristic

The formation and symmetry of the vortex within the GVWT basin were evaluated through CFD simulations for two configurations, namely Basin A and Basin B. For both configurations, the basin was divided into several data collection sections to enable a detailed analysis of the free‐surface profile and internal flow structure, as given in Figure 1b. The reference position *z*/*H* = 0 was defined at the base of the intake channel, and the same coordinate system was used consistently throughout the basin analysis, including the single‐stage runner configuration.

As shown in Figure 4, the basin configuration had a pronounced effect on vortex development. Basin A produced an asymmetric vortex, with the vortex core displaced from the basin centerline. This off‐center structure indicates that the incoming flow was not distributed uniformly within the basin, leading to an uneven rotational pattern. In contrast, Basin B generated a more centered and vertically coherent vortex. The installation of the 19° inlet‐channel notch angle improved the alignment of the incoming tangential flow, thereby reducing radial asymmetry and promoting a more stable vortex structure. This observation is consistent with previous experimental and numerical studies that have shown that tangential inlet alignment enhances vortex formation and reduces radial asymmetry [34].

**Figure 4:**
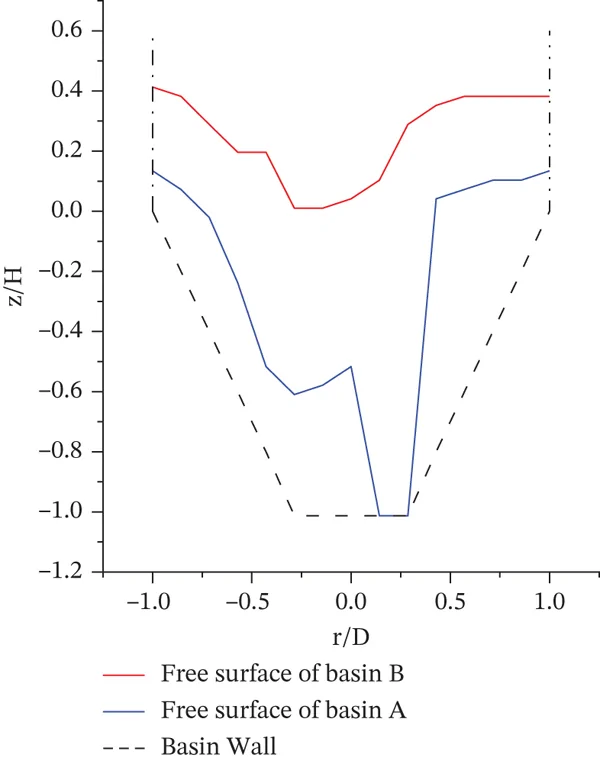
Free surface profile of the vortex.

A clear difference was also observed in the free‐surface profile. In Basin A, the air core extended over a relatively wide region, particularly in the upper part of the basin. This condition suggests a larger low‐water‐fraction zone near the vortex center, which may reduce the effective flow area available for energy transfer. By comparison, Basin B formed a taller vortex column with a more confined core region, indicating a stronger and more organized swirling motion. The higher water level observed in the inlet channel of Basin B also implies a greater available head, which contributes to the improved vortex development.

The more symmetric and vertically stable vortex in Basin B is advantageous for turbine operation because it provides a more uniform hydrodynamic condition at the runner plane. An asymmetric vortex, such as that observed in Basin A, may result in nonuniform velocity distribution and unequal hydraulic loading on the runner blades. In contrast, the improved vortex coherence in Basin B is expected to support more consistent blade immersion and a more stable transfer of momentum from the rotating flow to the runner.

The reduction in air‐core diameter at deeper sections further indicates that the flow becomes increasingly water‐dominated toward the lower part of the basin. This behavior is important because the usable vortex region for runner placement should not only possess sufficient tangential velocity but also maintain adequate blade submergence. The results demonstrate that Basin B significantly improved vortex symmetry and vertical coherence, thereby creating more favorable hydrodynamic conditions for energy extraction. These findings provide the basis for the subsequent analysis of velocity distributions in the two basin configurations.

These differences in vortex symmetry and free‐surface development indicate that the two basin configurations generate different internal flow structures. To clarify how the inlet modification affects the hydrodynamic conditions relevant to turbine operation, the tangential, axial, and radial velocity distributions are further examined in the next subsection.

### 3.2. Velocity Distribution in the Basin

To further explain the hydrodynamic differences between the two basin configurations, the tangential, axial, and radial velocity distributions were analyzed at several depths inside the basin. In this study, each velocity component (*v*_*t*_, *v*_*a*_, and *v*_*r*_) was normalized by the inlet velocity *V*, measured in the inlet channel. The reported profiles represent the average normalized velocity on the left and right sides of the basin. This analysis was intended to clarify how the inlet modification affects the rotational strength, vertical transport, and radial stability of the vortex, all of which are directly relevant to runner performance and basin energy concentration.

#### 3.2.1. Tangential Velocity

The tangential velocity component governs the angular momentum available for runner rotation and is therefore one of the most important indicators of GVWT performance. As shown in Figure 5, Basin A exhibited a strongly asymmetric tangential velocity distribution between the left and right sides of the basin, particularly at intermediate depths. At several locations, the discrepancy reached approximately 50%, indicating an uneven rotational flow structure. Although Basin A produced relatively high peak values of *v*_*t*_/*V*, reaching about 3 near *z*/*H* = −0.6, the flow was poorly distributed across the basin.

**Figure 5:**
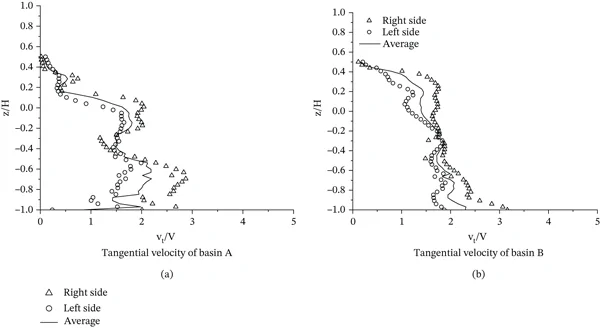
Profile of tangential velocity. Tangential velocity of (a) Basin A and (b) Basin B.

By contrast, Basin B showed a more uniform tangential velocity profile. The peak value of *v*_*t*_/*V* was lower, approximately 2.2 at *z*/*H* = −0.6, but the left–right asymmetry was reduced to less than 16%. This result indicates that Basin B improved the alignment of the incoming flow and promoted a more axisymmetric vortex. From a hydrodynamic perspective, this more uniform tangential distribution is advantageous because it is expected to provide a more even momentum transfer to the runner and a more stable torque response. This improved symmetry aligns with the findings of Janjua et al. [31], who reported that tangential flow uniformity can improve runner efficiency by ensuring a more consistent angle of attack across the blade span. Therefore, although Basin A exhibited higher local tangential velocity peaks, Basin B provided a more favorable flow structure for practical turbine operation.

#### 3.2.2. Axial Velocity

The axial velocity component reflects the vertical transport of flow toward the outlet and affects the loading condition along the runner depth. Basin A was characterized by abrupt variations in *v*_*a*_/*V*, particularly near the upper part of the basin (*z*/*H* = −0.05 to −0.2), as shown in Figure 6. These fluctuations indicate a less controlled inflow development and suggest that the flow entered the vortex core in a nonuniform manner. The axial asymmetry remained visible over a large portion of the basin depth, indicating that the vertical flow structure was less stable.

**Figure 6:**
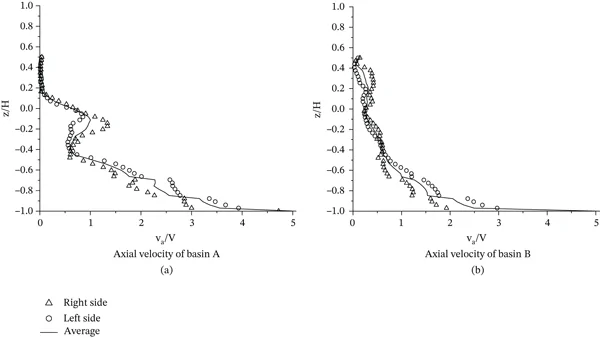
Profile of the axial velocity. Axial velocity of (a) Basin A and (b) Basin B.

In Basin B, the axial velocity profile was smoother and more symmetric. At the runner‐relevant depth of approximately *z*/*H* = −0.6, Basin B produced a lower axial velocity ratio, around 1.0, compared with about 1.75 in Basin A. This reduction suggests that the Basin B moderated the downward flow intensity and reduced abrupt axial variations. Such a flow structure is preferable because it indicates a more stable hydrodynamic environment and a lower likelihood of excessive localized axial loading on the runner. However, the smoother profile in Basin B may also imply a slight reduction in vertical flow intensity near the lower region of the basin, which should be considered in runner design.

#### 3.2.3. Radial Velocity

Although the radial velocity component does not directly contribute to torque generation, it provides useful information on secondary flow behavior and vortex coherence. A large radial component generally indicates stronger outward or inward flow deviations, which may reduce the effectiveness of rotational energy concentration. In Basin A, the radial velocity increased gradually with depth and approached values close to 1.0 near the outlet, as given in Figure 7. This behavior suggests stronger radial flow redistribution and a less confined vortex structure.

**Figure 7:**
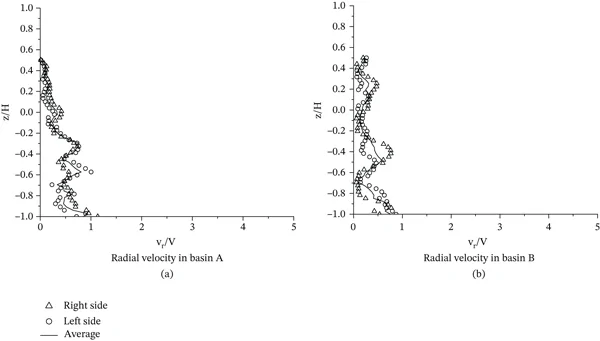
Profile of the radial velocity. Radial velocity in (a) Basin A and (b) Basin B.

In contrast, Basin B maintained a flatter radial velocity profile, with an average *v*_*r*_/*V* of about 0.75 and lower variation along the basin depth. This result indicates that the inlet‐channel notch angle not only improved tangential flow organization but also helped preserve vortex coherence by limiting radial divergence. The lower radial fluctuation in Basin B therefore supports the formation of a more stable and concentrated rotating flow [38].

The comparison of the three velocity components reveals a clear difference between the two basin configurations. Basin A tended to produce higher peak velocities in some regions, but the flow structure was less symmetric and less stable. Basin B, on the other hand, generated a more integrated hydrodynamic pattern: a sufficiently strong tangential velocity for torque generation, a smoother axial flow, and a more controlled radial component. These combined characteristics indicate that the inlet‐channel notch angle improved not only vortex formation, but also the internal flow quality relevant to turbine operation. This higher quality flow structure is particularly important for runner operation and provides a useful basis for evaluating the feasibility of multistage energy extraction.

#### 3.2.4. Nondimensional Velocity Formulation

To further interpret the internal flow behavior in the conical GVWT basin, a nondimensional analysis was performed by relating the basin geometry, flow rate, and vertical position to the three primary velocity components. The derived empirical relationships are given by Equations (2)–(4):

(2) vt=2.25∙Qz2Dz−2tanθ−0.75,

(3) va=1.19∙Qz2Dz−2tanθ−0.53,

(4) vr=0.49∙Qz2Dz−2tanθ−1.5,

where *v*_*t*_, *v*_*a*_ and *v*_*r*_ are tangential, axial, and radial velocities (m/s), respectively; *Q* is the flow rate (m^3^/s). *z* is the vertical position at which the velocity is evaluated (m); *D* is basin upper diameter (m); and *θ* is the cone angle ( ^°^).

These relationships show that all three velocity components decrease as the geometric ratio increases, which is physically consistent with the expansion of the basin cross‐section toward the upper region. As the effective radius becomes larger, the rotational and vertical momentum are diluted, whereas deeper regions with smaller cross‐sectional area promote higher local kinetic energy density. The stronger exponent in the radial velocity equation indicates that *v*_*r*_ decays more rapidly than *v*_*t*_ and *v*_*a*_, suggesting that radial motion is more sensitive to geometric expansion. This is hydrodynamically favorable because it means that the flow becomes increasingly vortex‐dominated as it moves toward the lower part of the basin.

This interpretation is further supported by the velocity ratios shown in Figure 8. The ratios *v*_*t*_/*v*_*r*_ and *v*_*t*_/*v*_*a*_ reached their maximum values at approximately *z*/*H* = −0.7, with values of about 20 and 12.7, respectively. These high ratios indicate that the flow in this region was dominated by tangential motion while radial diffusion and excessive axial dominance remained limited. Such a condition is highly favorable for turbine operation because it suggests that the available hydraulic energy is concentrated in the direction most relevant to runner torque generation.

**Figure 8:**
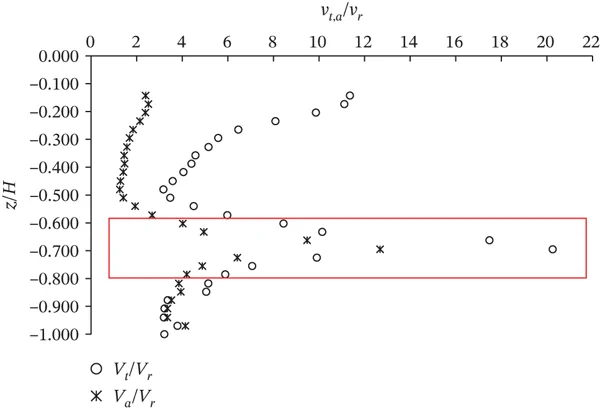
Ratios of tangential and axial velocity.

The velocity analysis confirms that Basin B provides a more organized and hydrodynamically favorable vortex structure than Basin A. The improved flow symmetry, smoother axial development, and reduced radial diffusion establish the basis for the subsequent evaluation of power prediction and the identification of a suitable region for multistage runner placement. Because the observed velocity structure governs the hydrodynamic energy available to the runner, the next subsection evaluates its relationship to the predicted power output through a semiempirical model.

#### 3.2.5. Velocity Triangle Analysis

To further interpret the hydrodynamic condition at the runner plane, a schematic velocity‐triangle diagram was constructed, as shown in Figure 9. The diagram represents the decomposition of the local incoming flow into axial and tangential components, together with the corresponding inlet flow angle (*α*). This schematic is intended to support the interpretation of the flow entering the propeller runner and to illustrate that the incoming flow contains both axial and tangential components relevant to energy extraction. However, the present study does not perform a complete blade‐angle optimization, and the velocity triangle is therefore used here as a hydrodynamic interpretation tool rather than as a full runner‐design procedure. The value of inlet flow angle is given by Table 3.

**Figure 9:**
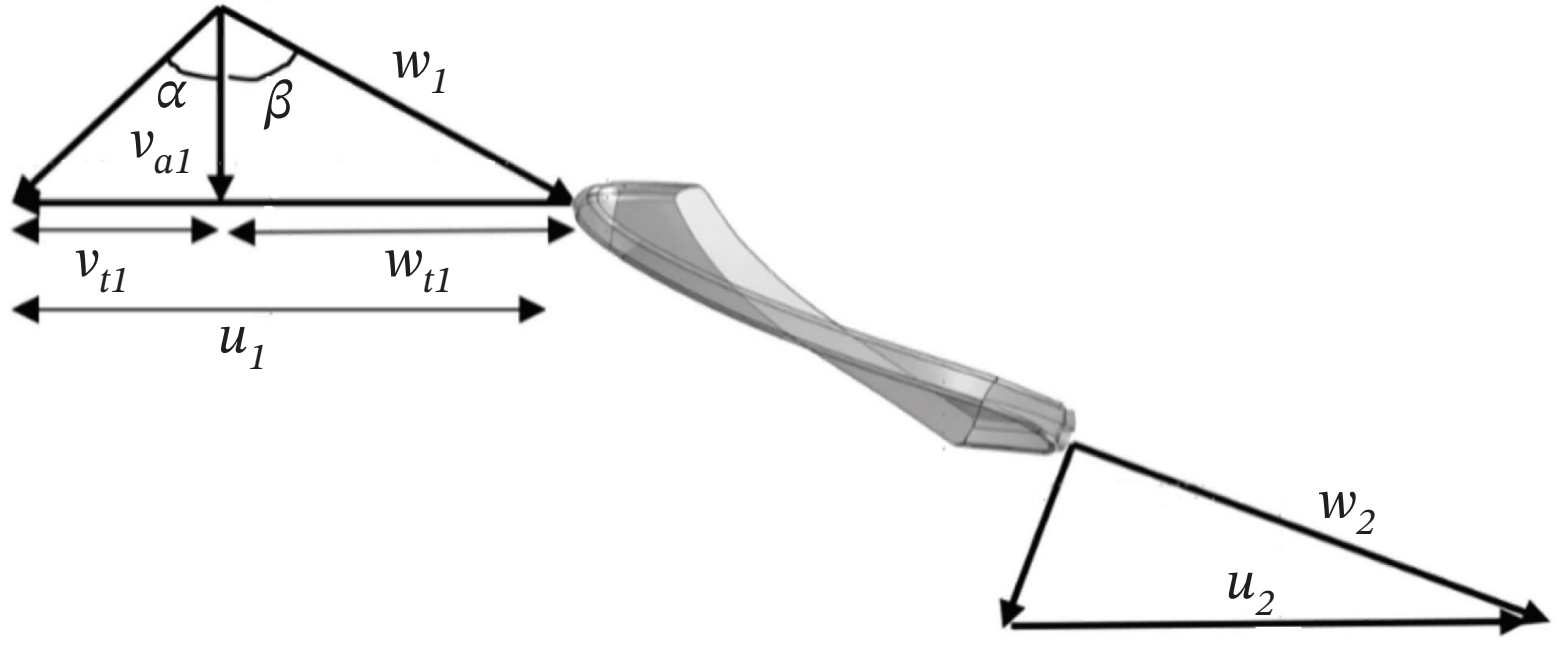
Schematic velocity‐triangle diagram at the runner plane, illustrating the axial velocity component *v*_*a*1_, tangential velocity component *v*_*t*1_, blade velocity *u*, relative velocity *w*, and the inlet flow angle *α*.

**Table 3:** Estimated inlet flow angle *α* at different runner installation depths based on the local tangential and axial velocity components.

z/H	Inlet flow angle	
	Basin A	Basin B
−0.5	67.6	71.7
−0.6	62.4	72.5
−0.7	57.0	66.1
−0.8	54.2	63.0

### 3.3. Power Prediction Model Performance

To provide a practical design tool, a semiempirical model was developed to predict the power output of the single‐stage GVWT. The predicted power is expressed as

(5) P=ρQ3ηDr4358.07664.48317.24zH2−zH+D−2ztanθDr−6,

where *P* is the turbine power (W), *ρ* is the fluid density, *Q* is the discharge, *D*_*r*_ is the runner diameter, and *z*/*H* is the nondimensional runner installation depth. *z* refers to the vertical position of the runner midplane, measured relative to the inlet‐channel bottom level, which was defined as (*z*/*H* = 0). Negative values of (z/H) indicate that the runner is located below this reference level. *d* is the upper basin diameter and *θ* is the cone angle. The efficiency of GVWT (*η*) for *z*/*H* −0.6, −0.7, and −0.8 are 0.29, 0.28, and 0.21, respectively. The efficiency values (*η*), were obtained from the authors′ experimental GVWT performance tests. Therefore, the efficiency term in the present model represents an experimentally derived, depth‐dependent parameter rather than an assumed constant value.

The predictive capability of Equation (5) was evaluated using experimental data for a 10 cm propeller runner installed at *z*/*H* = −0.6, −0.7, and −0.8. The model was formulated using CFD‐derived hydrodynamic variables and then evaluated against the experimental power data. The model reproduced the experimental trend reasonably well, confirming that runner depth has a strong influence on the extractable power. Good agreement was obtained at *z*/*H* = −0.8 and *z*/*H* = −0.6, where the deviation was about 2%, as shown in Figure 10. This indicates that the hydrodynamic formulation is able to capture the depth‐dependent power trend, but its accuracy is sensitive to the efficiency value adopted in the prediction.

**Figure 10:**
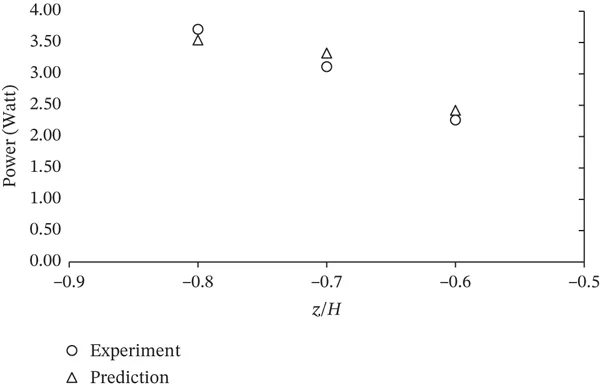
Comparison between experimental and predicted power at different runner installation depths.

For the power‐prediction validation, the uncertainty was evaluated by considering the propagation of the main measured variables used to calculate experimental torque and power, including tangential force of the runner, rotational speed, and total head. The uncertainty of tangential force, rotational speed, and total head of the experimental data are*u*_*FT*_ = ±0.083 N,*u*_*ω*_ = 0.26 rad/s, and*u*_*l*_ = 0.0005 m, respectively. The propagation uncertainty of torque and power are *u*_*τ*_ = ±1.9*%* and *u*_*p* *o* *w* *e* *r*_ = ±2.2*%*, respectively.The resulting uncertainty range indicates whether the proposed semiempirical model for power prediction reproduces the experimental trend within the expected uncertainty range of the measurements.

The proposed model provides a reasonable estimate of GVWT power output and is suitable for preliminary design evaluation. However, a depth‐dependent efficiency term would be required to improve its predictive accuracy over a wider range of runner positions. With the predictive capability of the model established, the remaining downstream flow is further analyzed to identify the hydrodynamic basis for multistage runner placement.

In the present study, the experimental and numerical datasets were obtained using the same conical basin geometry, where basin inlet diameter, basin outlet diameter, basin height, and cone angle were treated as constant geometric parameters in the calibrated semiempirical model. Consequently, the proposed model should be interpreted as a geometry‐specific correlation for the tested GVWT configuration rather than a universal power‐prediction equation for arbitrary basin geometries.

### 3.4. Hydrodynamic Basis for Multistage Runner Placement

The feasibility of a multistage GVWT depends strongly on the amount and form of the hydraulic energy that remains downstream of the first‐stage runner, as well as on the local hydrodynamic quality of the residual flow. For this reason, the kinetic‐energy distribution before and after runner installation was analyzed to determine whether the flow below the first stage still retains sufficient hydrodynamic quality for additional energy extraction.

Figure 11 compares the total head and total kinetic energy profiles for the basin without a runner and the single‐stage GVWT configuration. Before runner installation, the basin exhibited a higher kinetic‐energy profile above the runner plane because the flow remained unobstructed and the velocity field was not affected by energy extraction. After installation of the first‐stage runner at *z*/*H* = −0.5, the flow experienced a clear redistribution of energy. In the single‐stage configuration, kinetic energy decreased above the runner because of flow resistance and partial energy extraction but increased again immediately below the runner, particularly around *z*/*H* = −0.6. This local buildup is associated with the combined effect of basin contraction and the persistence of swirling motion after passing through the runner. The result indicates that the downstream flow is not merely residual discharge, but still contains usable hydraulic energy that can be evaluated for second‐stage runner placement.

**Figure 11:**
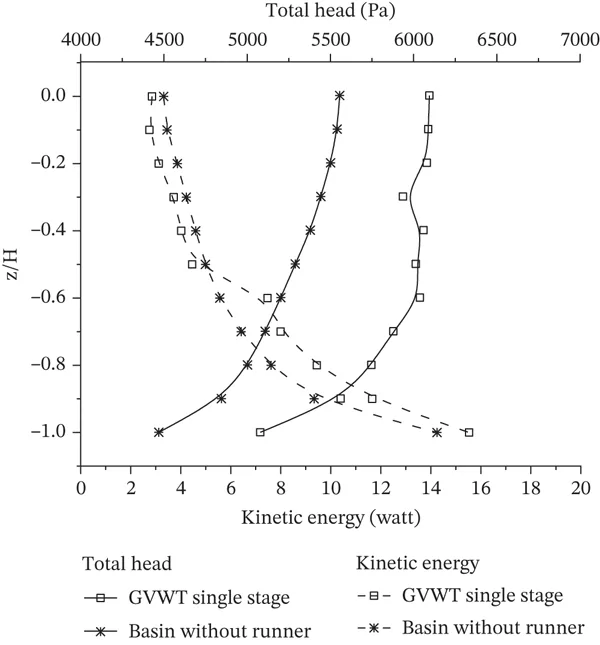
Kinetic energy and total head of the basin without runner and the GVWT single‐stage configuration.

A more detailed interpretation is obtained by separating the kinetic energy into its tangential and axial components, as shown in Figure 12. The tangential kinetic energy, presented in Figure 12a, is the dominant component throughout most of the basin depth and therefore provides the primary basis for assessing second‐stage runner placement because it represents the residual rotational energy available for torque generation. Below the first‐stage runner, the tangential kinetic energy initially remains high and reaches a local maximum near *z*/*H* = −0.6, confirming that strong rotational motion is still preserved after the first extraction stage. However, it then decreases markedly around *z*/*H* = −0.7, indicating a local region of energy depletion where immediate second‐stage placement would be less favorable. Further downstream, between *z*/*H* = −0.8 and −0.9, the tangential kinetic energy becomes more stable, suggesting that the vortex has regained a more coherent downstream structure and that the residual rotational energy is more uniformly distributed. This region is therefore more suitable for second‐stage runner installation because it combines high residual tangential energy with improved downstream flow coherence.

**Figure 12:**
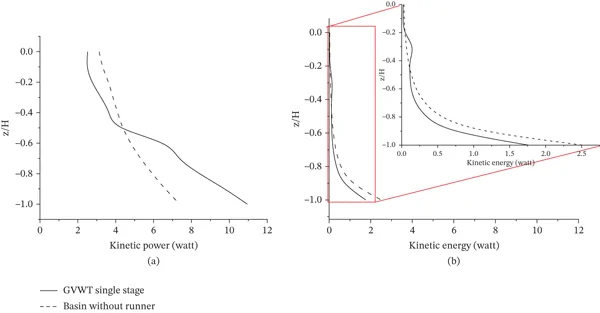
Profiles of (a) tangential kinetic energy and (b) axial kinetic energy.

The axial kinetic energy, shown in Figure 12b, exhibits a different trend. It increases more rapidly in the upper region of the basin and near the runner plane, reflecting the downward acceleration of the flow toward the outlet.

Beyond the first‐stage runner, the axial component remains appreciable but does not dominate the energy distribution to the same extent as the tangential component, indicating that the downstream flow remains vortex‐dominated rather than purely jet‐dominated. This is an important observation because a second‐stage runner should ideally be placed where the flow remains vortex‐dominated rather than purely jet‐dominated. In the lower basin, the coexistence of sustained tangential kinetic energy and moderate axial kinetic energy indicates that the downstream flow still possesses a favorable balance for additional torque generation without excessive axial dominance.

These energy trends are consistent with the velocity profiles discussed in the previous subsection, where the lower basin was shown to retain strong tangential motion, reduced radial diffusion, and improved submergence conditions. The lower part of the basin retains strong tangential motion, moderate axial energy, and improved submergence conditions, which together define the main hydrodynamic criteria for second‐stage runner placement. Therefore, the recommended region for the second‐stage runner is *z*/*H* = −0.9 to −0.8, where the residual tangential kinetic energy remains high, the axial component stays moderate, radial diffusion is reduced, and the runner can be installed under more favorable submergence and geometric‐clearance conditions.

The installation of the first‐stage runner significantly modifies the downstream energy distribution but does not eliminate the hydrodynamic potential of the lower basin. Instead, it creates a depth‐dependent redistribution of kinetic energy that must be considered carefully in multistage design. The present results show that second‐stage runner placement should be determined not by geometric spacing alone but by explicit hydrodynamic criteria. In this respect, the region *z*/*H* = −0.9  to −0.8 provides the most favorable hydrodynamic basis for multistage runner placement in the present conical GVWT configuration and may serve as a useful design reference for future multistage GVWT studies. Although the region *z*/*H* = −0.9  to −0.8  provides favorable residual hydrodynamic characteristics for second‐stage energy extraction, the runner diameter must be selected by considering the local basin diameter and the required blade‐tip clearance. Therefore, the proposed second‐stage placement should be interpreted together with a runner‐size constraint, and a smaller runner diameter is required for installation in the lower basin region.

Despite the robust alignment between the numerical and experimental results, it is important to acknowledge certain limitations in the current validation scope. The experimental validation in this study was primarily focused on the macroscopic free‐surface profile, which showed an average deviation of 5%. Although direct internal velocity measurements, such as through particle image velocimetry (PIV) or Pitot tubes, were not conducted, the high predictive accuracy of the power model at and serves as a reliable proxy for the validity of the internal flow field. Future research incorporating high‐resolution internal flow measurements would further refine the understanding of localized turbulence and its impact on efficiency, particularly in more complex multistage interactions.

## 4. Conclusion

This study investigated the hydrodynamic behavior of two conical basin configurations, Basin A (inlet‐channel notch angle is 0°) and Basin B (inlet‐channel notch angle is 19°), in order to develop a design‐oriented interpretation of how basin geometry affects performance prediction and multistage runner placement. Compared with Basin A, Basin B produced a more uniform tangential velocity distribution, a smoother axial velocity profile, and reduced radial diffusion, indicating a more favorable hydrodynamic environment for energy extraction. For the single‐stage configuration, the runner is recommended to be installed around (*z*/*H* = −0.7), where the tangential velocity is relatively high and favorable for energy extraction.

A semiempirical power prediction model derived from Buckingham pi analysis was able to reproduce the experimental power trend reasonably well, with deviations of about 2%. The downstream flow analysis showed that the lower part of the basin still retained high residual tangential energy, moderate axial energy, and favorable submergence conditions after the first‐stage runner, thereby providing a practical hydrodynamic basis for second‐stage runner placement. If the first stage runner is placed in *z*/*H* = −0.5, the region *z*/*H* = −0.9 to −0.8 is the most suitable for second‐stage runner installation. However, the runner diameter must be smaller than that of the first stage because of the reduced local basin diameter and the required blade‐tip clearance.

Future work should further investigate the pressure distribution and pressure drop across the runner blades to provide a more detailed explanation of the energy‐conversion mechanism. Such analysis would help clarify the relationship between local pressure variation, torque generation, angular‐momentum transfer, and the overall performance of single‐stage and multistage GVWT systems.

**Nomenclature**
*D*_*b*_(*z*) : local basin diameter at vertical position z, particularly at the runner installation plane, *m*
*D*_*o*_
: bottom outlet diameter of the basin, *m*
*D*_*r*_
: runner diameter, *m*
D
: upper diameter of the conical basin, *m*
*F*_*t*_
: tangential force acting on the runner, *N*
g
: gravitational acceleration, *m* *s* ^−2^
H
: basin height used as the reference length for nondimensional depth, *m*
P
: turbine power output, *W*
*P*_*p* *r* *e* *d*_
: predicted turbine power output from the semiempirical model, *W*
Q
: water flow rate or discharge, *m* ^3^ *s* ^−1^
r
: local radial position in the basin, *m*
τ
: torque generated by the runner, *N*∙*m*
V
: inlet velocity used for velocity normalization, *m* *s* ^−1^
*v*_*a*_
: axial velocity component, *m* *s* ^−1^
*v*_*r*_
: radial velocity component, *m* *s* ^−1^
*v*_*t*_
: tangential velocity component, *m* *s* ^−1^
*v*_*a*_/*v*
: normalized axial velocity, dimensionless
*v*_*r*_/*V*
: normalized radial velocity, dimensionless
*v*_*t*_/*V*
: normalized tangential velocity, dimensionless
*v*_*t*_/*v*_*a*_
: ratio of tangential velocity to axial velocity, dimensionless
*v*_*t*_/*v*_*r*_
: ratio of tangential velocity to radial velocity, dimensionless
z
: vertical position of the observation plane or runner midplane measured relative to the inlet‐channel bottom level, *m*
*z*/*H*
: nondimensional vertical position or runner installation depth, dimensionless
η
: turbine efficiency, dimensionless
θ
: cone angle of the basin,  ^°^
μ
: dynamic viscosity of water, *P* *a*∙*s*
ρ
: density of water, *k* *g* *m* ^−3^
ω
: angular velocity of the runner, *r* *a* *d* *s* ^−1^

**Abbreviations**
CFD: computational fluid dynamics
CFX: computational fluid dynamics software module used in ANSYS CFX
GVWT: gravitational vortex water turbine
*k* − *ε*
: turbulent kinetic energy–dissipation rate turbulence model
LHP: low‐head hydropower
PAT: pump‐as‐turbine
PIV: particle image velocimetry
RNG: Renormalization Group
RPM: revolutions per minute
ULH: ultralow head
VOF: volume of fluid

## Funding

This work was supported by the Indonesian Endowment Fund for Education (LPDP), 3029/PKS/ITS/2025.

## Conflicts of Interest

The authors declare no conflicts of interest.

## Data Availability

The data that support the findings of this study are available from the corresponding author upon reasonable request.
